# Astaxanthin: A Potential Therapeutic Agent in Cardiovascular Disease

**DOI:** 10.3390/md9030447

**Published:** 2011-03-21

**Authors:** Robert G. Fassett, Jeff S. Coombes

**Affiliations:** 1 Renal Research Royal Brisbane and Women’s Hospital and The University of Queensland School of Medicine, Level 9 Ned Hanlon Building, Butterfield Street, Brisbane, Queensland 4029, Australia; 2 School of Human Movement Studies, The University of Queensland, St. Lucia, Brisbane, Queensland 4072, Australia; E-Mail: jcoombes@uq.edu.au

**Keywords:** antioxidants, xanthophyll carotenoid, inflammation, *Haematococcus pluvialis*, oxidative stress

## Abstract

Astaxanthin is a xanthophyll carotenoid present in microalgae, fungi, complex plants, seafood, flamingos and quail. It is an antioxidant with anti-inflammatory properties and as such has potential as a therapeutic agent in atherosclerotic cardiovascular disease. Synthetic forms of astaxanthin have been manufactured. The safety, bioavailability and effects of astaxanthin on oxidative stress and inflammation that have relevance to the pathophysiology of atherosclerotic cardiovascular disease, have been assessed in a small number of clinical studies. No adverse events have been reported and there is evidence of a reduction in biomarkers of oxidative stress and inflammation with astaxanthin administration. Experimental studies in several species using an ischaemia-reperfusion myocardial model demonstrated that astaxanthin protects the myocardium when administered both orally or intravenously prior to the induction of the ischaemic event. At this stage we do not know whether astaxanthin is of benefit when administered after a cardiovascular event and no clinical cardiovascular studies in humans have been completed and/or reported. Cardiovascular clinical trials are warranted based on the physicochemical and antioxidant properties, the safety profile and preliminary experimental cardiovascular studies of astaxanthin.

## 1. Introduction

Astaxanthin is a xanthophyll carotenoid of predominantly marine origin, with potent antioxidant and anti-inflammatory effects demonstrated in both experimental and human studies. Oxidative stress and inflammation are common pathophysiological features of atherosclerotic cardiovascular disease hence astaxanthin may have a potential therapeutic role in this condition. This review will summarise the available evidence suggesting astaxanthin may be of therapeutic value in cardiovascular disease.

## 2. Oxidative Stress and Inflammation

Oxidative stress and inflammation are established non-traditional risk factors for atherosclerosis associated cardiovascular morbidity and mortality [1]. Dietary antioxidants can reduce the oxidation of lipids and proteins and have the potential to protect from the development of arterial stiffening and atherosclerosis [2–4]. Cross-sectional and prospective observational studies have demonstrated an association between the intake of dietary antioxidants and/or their plasma levels and a reduction of cardiovascular events [5–10]. This supports the theory that oxidative stress is a pathophysiological process involved in atherosclerotic vascular damage. Also, a reduced dietary antioxidant intake is associated with oxidative stress and inflammation [11]. Newer more potent dietary antioxidants such as astaxanthin have yet to be studied in this setting. Studies that have assessed the intake of β-carotene or dietary β-carotene supplementation have shown higher β-carotene consumption is associated with a reduction in cardiovascular disease [6,12–17]. Other than a few studies [18–20], cardiovascular intervention trials using antioxidants have not demonstrated benefits [21–23]. This may be because study participants did not have oxidative stress and/or the antioxidants used were insufficiently potent. In addition, it is becoming recognized that there is communication between oxidative stress and inflammatory processes leading to the additional hypothesis that antioxidants may be able to modify both deleterious events. Further research is needed studying antioxidants with different biological actions in patients with demonstrated oxidative stress.

## 3. Carotenoids

Carotenoids are ubiquitous, and found in high concentrations in plants, algae and microorganisms. Humans and other animals cannot synthesize them and therefore are required to source them in their diet [24]. Carotenoids are classified, according to their chemical structure, into carotenes and xanthophylls. The carotene carotenoids include β-carotene and lycopene and the xanthophyll carotenoids include lutein, canthaxanthin, zeaxanthin, violaxanthin, capsorubin and astaxanthin [25,26].

The effects of carotenoids vary dependent on how they interact with cell membranes [25]. The effects of astaxanthin, zeaxanthin, lutein, β-carotene and lycopene on lipid peroxidation have been assessed using a polyunsaturated fatty acid enriched membrane model [25,27]. Non-polar carotene carotenoids such as lycopene and β-carotene caused membrane disorder and lipid peroxidation in contrast to the polar xanthophyll carotenoid astaxanthin, which preserved membrane structure [27]. Contrasting effects of different carotenoids may be responsible for the differing biological effects seen in clinical studies. For instance, in some studies the non-polar carotenoid, β-carotene has been shown to have no benefit on cardiovascular disease [28–32] and in fact it may be pro-oxidant at high doses [33]. In contrast, the polar carotenoid astaxanthin has protective effects on the cardiovascular system demonstrated in animal studies. However, this has not been studied in human clinical trials [34–36]. β-carotene at physiological levels may act in differing ways when ultraviolet A light A (UVA) acts on keratinocytes including vitamin A-independent pathways [37]. Astaxanthin, canthaxanthin and β-carotene had differential effects on UVA-induced oxidative damage [38]. In addition, carotenoids may also alter the immune response [39] and transcription [40].

## 4. Astaxanthin

**Figure 1.**
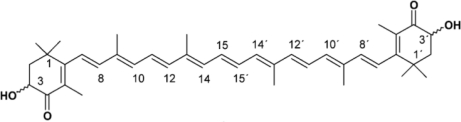
Molecular structure of astaxanthin.

Astaxanthin contains two oxygenated groups on each ring structure (see Figure 1), which is responsible for its enhanced antioxidant features [41]. It is found in living organisms particularly in the marine environment where it is present in microalgae, plankton, krill and seafood. It gives salmon, trout, and crustaceans such as shrimp and lobster their distinctive reddish coloration [42]. It is also present in yeast, fungi, complex plants and the feathers of some birds including flamingos and quail [42]. In 1987, the United States Food and Drug Administration approved astaxanthin as a feed additive for use in the aquaculture industry and in 1999 it was approved for use as a dietary supplement (nutraceutical) [41]. The microalgae *Haematococcus pluvialis* produces the astaxanthin isomer (3*S*, 3*S*′), which is the same as the form found in wild salmon. Synthesis of astaxanthin is not possible in humans and it cannot be converted to vitamin A, which means excess intake will not cause hypervitaminosis A toxicity [43,44]. Astaxanthin and canthaxanthin are scavengers of free radicals, chain-breaking antioxidants and potent quenchers of reactive oxygen and nitrogen species including singlet oxygen, single and two electron oxidants [45–47]. They (astaxanthin and canthaxanthin) have terminal carbonyl groups that are conjugated to a polyene backbone [26] and are more potent antioxidants and scavengers of free radicals than carotene carotenoids such as β-carotene [47,48]. For these reasons dietary supplementation with astaxanthin has the potential to provide antioxidant protection of cells and from atherosclerotic cardiovascular disease [49].

## 5. Astaxanthin Formulations

### 5.1. Astaxanthin of Marine Origin

Astaxanthin used in nutritional supplements is usually a mixture of configurational isomers produced by *Haematococcus pluvialis*, a unicellular microalga [50]. Astaxanthin can be produced in its natural forms on a large scale [51]. The initial production of astaxanthin from *Haematococcus pluvialis* uses closed culture systems followed by a 5–7 day, “reddening” cycle, conducted in open culture ponds. At each production stage, the cultures are closely monitored by microscopic examination to ensure they remain free of contamination. After the reddening cycle, *Haematococcus pluvialis* cultures are harvested, washed and dried. The final step for the production of astaxanthin is extraction of dried *Haematococcus pluvialis* biomass using supercritical carbon dioxide to produce a purified oleoresin, which is free of any contamination. Other sources used for the commercial production of astaxanthin include cultures of *Euphausia pacifica* (Pacific krill), *Euphausia superba* (Antarctic krill), *Pandalus borealis* (shrimp) and *Xanthophyllomyces dendrorhous*, formerly *Phaffia rhodozyma* (yeast). Astaxanthin from natural sources varies considerably from one organism to another. For instance, the astaxanthin found in seafood will depend on the stereoisomer ingested. Astaxanthin produced by *haematococcus pluvialis*, consists of the (3-*S*,3′-*S*) stereoisomer which is most commonly used in aquaculture. It is therefore the form most commonly consumed by humans.

### 5.2. Synthetic Astaxanthin

There are three stereoisomers of astaxanthin; (3-*R*,3′-*R*), (3-*R*,3′-*S*) and (3-*S*,3′-*S*). Disodium disuccinate astaxanthin (DDA) is a synthetic astaxanthin containing a mixture of all three stereoisomers, in the proportions 1:2:1. DDA was manufactured by Cardax Pharmaceuticals and used in animal studies investigating the myocardial ischemia-reperfusion injury models [34–36,52–54]. This form of astaxanthin was touted to have better aqueous solubility, unlike other carotenoids, and this enabled both oral and intravenous administration. DDA is no longer available but the same company now produces a second synthetic astaxanthin compound; Heptax/XanCor, CDX-085. The company claims that it is developed for thrombotic protection, triglyceride reduction, metabolic syndrome, and inflammatory liver disease. In addition, it has increased water dispersibility and enhanced bioavailability compared to natural astaxanthin and DDA. The synthetic forms are metabolized via hydrolysis in the intestine yielding free astaxanthin for intestinal absorption. CDX-085 has been used in one study, discussed below [55].

It is not yet clear which form of astaxanthin should be administered in clinical studies, the natural form from the marine environment or a synthetic form. As the proportions of stereoisomers, vary between these different forms of astaxanthin they may not be therapeutically equivalent [56]. Thus synthetic astaxanthin could result in different outcomes when assessed clinically [57].

## 6. Astaxanthin-Experimental Studies

Experimental studies undertaken with astaxanthin specifically relevant to the cardiovascular system are summarised in Table 1. Astaxanthin attenuates mediators of oxidative stress and inflammation and has shown beneficial effects in non-cardiovascular models of disease [58–69]. In addition, astaxanthin has decreased markers of lipid peroxidation [70], inflammation [61,62,67,68] and thrombosis [55].

### 6.1. Cardiovascular Studies

A series of experiments have been conducted to assess the efficacy of DDA in protecting the myocardium using the myocardial ischemia-reperfusion model in rats, rabbits and dogs [35,36,53,54]. Prior treatment for four-days with intravenous DDA using doses of 25, 50 and 75 mg/kg body weight in Sprague-Dawley rats significantly reduced myocardial infarct size [35]. The degree of cardiac protection correlated with the dose of DDA administered. In a study in rabbits using a myocardial ischaemia-reperfusion model prior intravenous treatment with 50 mg/kg/day of DDA for four days resulted in a significant decrease in the size of the myocardial infarction and an improvement in myocardial salvage [52]. Animals treated with DDA had an attenuation of inflammation and complement activation suggesting there was a reduction in tissue inflammation [52]. In another study using a dog model intravenous DDA was administered daily for four-days prior to occlusion of the left anterior descending coronary artery or two hours prior to coronary artery occlusion [54]. After an hour of coronary occlusion and three hours of reperfusion there was a significant reduction in myocardial infarct size in the dogs treated with DDA. In the four-day treatment group, two out of three dogs had complete cardiac protection [54]. In a rat study, the effects of seven days of pre-treatment with oral DDA, 125 and 500 mg/kg/day on the concentrations of free astaxanthin in myocardial tissue [36]. The astaxanthin concentration in the myocardium was 400 nM after oral DDA at a dose of 125 mg/kg/day for seven-days and it was 1634 nM after 500 mg/kg/day. There was also a reduction of multiple lipid peroxidation products. The doses of DDA used in these experiments were quite high and at this stage it is not known whether such doses would be safe to use in humans.

The effects of astaxanthin on blood pressure (BP) were assessed in spontaneously hypertensive rats (SHR). There was a significant reduction in BP after 14-days of oral astaxanthin administration whereas this did not occur in normotensive Wistar Kyoto rats [71]. Astaxanthin administered orally for five-weeks in stroke prone SHR also resulted in a significant BP reduction [71]. Oral astaxanthin also enhanced nitric oxide induced vascular relaxation in the rat aortas [71] In experiments in SHR, oral astaxanthin significantly decreased nitric oxide end products indicating that it may be exerting its BP effects via this pathway [72]. Studies using the SHR aorta and coronary arteries demonstrated that astaxanthin reduced the wall/lumen ratio in coronary arteries and decreased elastin bands in the aorta [72]. This suggests that astaxanthin may beneficially mediate atherosclerotic CVD processes.

Recently, a series of two experiments were reported in the one article, one using the synthetic astaxanthin (CDX-085) and the other using free astaxanthin [55]. CDX-085 administered orally to C57BL/6 mice resulted in the presence of free astaxanthin in the plasma, heart, liver and platelets. Mice that were fed astaxanthin had significantly increased basal arterial blood flow and a delay in occlusive thrombosis after endothelial injury. Also, in an *in vitro* study, human umbilical vein endothelial cells and platelets isolated from Wistar-Kyoto rats that were treated with free astaxanthin has significantly increased nitric oxide release and a decrease in peroxynitrite levels [55]. The authors concluded the results support the potential of astaxanthin as a potential therapy to prevent thrombosis associated with cardiovascular disease.

Astaxanthin administered to C57BL/6 mice resulted in a reduction in exercise-induced increases in the oxidative stress biomarkers 8-hydroxy-2′-deoxyguanosine and 4-hydroxy-2-nonenal-modified protein in both cardiac and gastrocnemius muscle [63]. Increases in myeloperoxidase and creatinine kinase activity in cardiac and gastrocnemius muscle were also reduced by astaxanthin. After three-weeks of astaxanthin supplementation there was evidence of accumulation of astaxanthin in gastrocnemius and cardiac muscle. Astaxanthin given to female BALB/c mice for eight-weeks resulted in a dose dependent increase in plasma astaxanthin but no significant changes in blood glutathione or change in lymphocyte mitochondrial membrane potential and cardiac contractility index measured on echocardiography. The mice that were fed 0.08% astaxanthin in the diet had higher cardiac mitochondrial membrane potential and contractility index compared with control animals [74]. This suggests dietary astaxanthin provides cardiac protection. Astaxanthin administered for four weeks to eight week old ICR mice resulted in increased exercised induced fat utilization and prevention of increased hexanoyl-lysine modification of carnitine palmitoyltransferase I (CTP I) [73]. In a canine carotid artery thrombosis model, administration of DDA resulted in a dose-dependent reduction in carotid artery re-thrombosis and a reduction of re-thrombosis after thrombolysis but there was no effect on hemostasis [34].

### 6.2. Diabetes Studies

Diabetes mellitus and its associated nephropathy is a common cause of chronic kidney disease and is complicated by accelerated atherosclerotic cardiovascular disease [75]. In studies involving diabetic db/db mice, supplementation with astaxanthin produced a reduction in the levels of blood glucose [60]. In the kidney there was significantly decreased relative mesangial area in the animals supplemented with astaxanthin. Also proteinuria and urinary excretion of 8-OHdG were attenuated. Mice supplemented with astaxanthin had less glomerular 8-OHdG immunoreactive cells [60]. Hyperglycemia induced reactive oxygen species production, activation of transcription factors, and cytokine expression and production by normal human mesangial cells was suppressed significantly by astaxanthin [66].

## 7. Astaxanthin Studies in Humans

Although no cardiovascular outcomes studies using astaxanthin have been reported in humans there have been clinical studies that have investigated the effects of astaxanthin in human health and other diseases (Table 2). The majority of these have been conducted in healthy participants who volunteered to assess astaxanthin dose, bioavailability, safety and oxidative stress, which are all potentially relevant to the cardiovascular system. Studies have also been conducted in other medical conditions such as reflux oesophagitis, where measurements of oxidative stress and/or inflammation have been included.

### 7.1. Dosing

Human clinical studies have used oral astaxanthin in a dose that ranges from 4 mg up to 100 mg/day, given from a one off dose up to durations of one-year (Table 2).

### 7.2. Bioavailability

Astaxanthin bioavailability from the marine environment was assessed in a randomised double blind trial in 28 volunteers [83]. Participants were given either 250 g of wild salmon or aquaculture salmon (5 μg/g) to eat. Wild salmon ingest astaxanthin naturally from krill whereas aquacultured salmon acquire it from fish that are fed astaxanthin that might be derived from a synthetic source. Plasma levels of astaxanthin were higher at 3, 6, 10 and 14 days during ingestion of the aquacultured compared with the wild salmon. Plasma levels of the (3-*S*, 3′-S) isomer of astaxanthin appeared at higher levels than its proportionate level in the flesh of the salmon. This suggests that isomers of astaxanthin might have different bioavailability. The plasma isomers of astaxanthin have also been studied after ingestion of single oral dose of 10mg and also 100 mg over four-weeks. Astaxanthin plasma elimination half-life was 52 (SD 40) h and there was a non-linear dose response and selective absorption of z-isomers [79].

### 7.3. Safety

The safety of astaxanthin administered orally was assessed in a double-blind, randomised placebo-controlled trial undertaken in healthy adults [78]. Volunteers took either 6 mg/day of astaxanthin or placebo for eight-weeks. BP and biochemistry measured after four and eight weeks of therapy revealed no significant differences in these parameters between treatment and placebo groups and these did not differ from baseline. The authors concluded that healthy adults could safely consume 6 mg/day of astaxanthin derived from a *Haematococcus pluvialis* algal extract. Measuring whole blood transit time in 20 healthy males was used to assess the effects of astaxanthin on blood rheology in humans. Six milligrams of oral astaxanthin per day for ten days improved blood rheology as evidenced by decreased whole blood transit time [82]. Escalating concentrations of astaxanthin were tested *in vitro* with blood taken from volunteers, 8 of whom were taking asprin and 12 who were not [85]. Even supra-therapeutic concentrations of astaxanthin had no adverse effects on indices of platelet, coagulation and fibrinolytic function. These results support the safety profile of astaxanthin for future clinical trials. No significant side effects have been reported so far in published human studies in which astaxanthin was administered to humans.

### 7.4. Oxidative Stress and Inflammation

Oral supplementation with astaxanthin in studies in healthy human volunteers and patients with reflux oesophagitis demonstrated a significant reduction in oxidative stress, hyperlipidemia and biomarkers of inflammation [70,80,86]. In a study involving 24 healthy volunteers who ingested astaxanthin in doses from 1.8 to 21.6 mg/day for two weeks, LDL lag time, as a measure of susceptibility of LDL to oxidation, was significantly greater in astaxanthin treated participants indicating inhibition of the oxidation of LDL [70]. Plasma levels of 12- and 15-hydroxy fatty acids were significantly reduced in 40 healthy non-smoking Finnish males given astaxanthin [80] suggesting astaxanthin decreased the oxidation of fatty acids [80]. The effects of dietary astaxanthin in doses of 0, 2 or 8 mg/day, over 8 weeks, on oxidative stress and inflammation were investigated in a double blind study in 14 healthy females [84]. Although these participants did not have oxidative stress or inflammation those taking 2 mg/day had lower CRP at week eight. There was also a decrease in DNA damage measured using plasma 8-hydroxy-2′-deoxyguanosine after week four in those taking astaxanthin. Astaxanthin therefore appears safe, bioavailable when given orally and is suitable for further investigation in humans.

## 8. Clinical Trial Using Astaxanthin

A double-blind randomised placebo-controlled clinical trial (Xanthin study) is currently being conducted to assess the effects of astaxanthin 8mg orally day on oxidative stress, inflammation and vascular function in patients that have received a kidney transplant [87]. Patients in the study undertake measurements of surrogate markers of cardiovascular disease including aortic pulse wave velocity, augmentation index, brachial forearm reactivity and carotid artery intima-media thickness. Depending on the results from this pilot study a large randomised controlled trial assessing major cardiovascular outcomes such as myocardial infarction and death may be warranted.

## 9. Conclusions

Experimental evidence suggests astaxanthin may have protective effects on cardiovascular disease when administered prior to an induced ischemia-reperfusion event. In addition, there is evidence that astaxanthin may decrease oxidative stress and inflammation which are known accompaniments of cardiovascular disease. At this stage we do not know whether astaxanthin has any therapeutic value in human cardiovascular disease either in a preventative capacity or when administered after a cardiovascular insult. It has been proposed that astaxanthin may provide cardiovascular protection through reducing oxidative stress, which is one of the non-traditional risk factors for the development of atherosclerotic cardiovascular disease. The role of oxidative stress in cardiovascular disease is supported by evidence from observational studies that have found associations between antioxidant intake, oxidative stress and cardiovascular outcomes. Despite this, clinical intervention studies using antioxidants including vitamin E, β-carotene and vitamin C, have not proved successful [22,23]. These intervention studies may have failed because of flawed design where patients were not included based on the presence of oxidative stress. Hence, many participants may not have been in a state of oxidative stress and able to benefit from antioxidant therapy. Also, in those participants where oxidative stress may have existed there was no way of assessing whether the therapy adequately corrected this. Thus, the antioxidants used such as vitamin E, β-carotene and vitamin C may not have been effective because insufficient doses were used or an inadequate length of therapy followed to correct the oxidative stress. Some antioxidants such as β-carotene may be pro-oxidant at higher doses, which could have confounded study results.

Astaxanthin is a potent antioxidant and based on its physicochemical properties and the results of preliminary experimental studies in ischaemia-reperfusion models of cardiovascular disease, it warrants consideration for testing in human clinical trials. There have been no safety concerns noted so far in human clinical studies where astaxanthin has been administered. As astaxanthin is a potent antioxidant and is associated with membrane preservation, it may protect against oxidative stress and inflammation and provide cardiovascular benefits.

## Figures and Tables

**Table 1.** Animal studies investigating the cardiovascular effects of astaxanthin.

**Study**	**Model**	**Dosage**	**Duration/timing of supplementation**	**Effects of astaxanthin**
Lauver *et al.* 2008 [34]	Dog with occlusive carotid artery thrombus	DDA 10, 30, or 50 mg/kg/body weight IV	30 min after occlusion	- Reduced incidence of secondary thrombosis
Aoi *et al.* 2003 [63]	C57BL/6 mice	Diet supplemented with astaxanthin 0.02% weight/weight and food intake recorded	3 weeks	- Attenuation of exercise increased 4-hydroxy-2-nonenal-modified protein and 8-hydroxy-2′-deoxyguanosine in cardiac and gastrocnemius muscle - Attenuation of exercise increases in creatine kinase and myeloperoxidase activity in cardiac and gastrocnemius muscle - Astaxanthin accumulated in cardiac and gastrocnemius muscle
Gross and Lockwood 2004 [35]	Myocardial infarct model Sprague-Dawley rats	DDA 25/50/75 mg/kg body weight intravenously daily	4 days prior to myocardial infarction	- Myocardial infarct size significantly reduced
Hussein *et al.* 2005 [71]	Stroke prone Spontaneously hypertensive rats	Astaxanthin 50 mg/kg body weight/day	5 weeks	- Significant blood pressure reduction - Delayed incidence of stroke
Lauver *et al.* 2005 [52]	Rabbit model of myocardial ischemia/reperfusion	DDA 50 mg/kg body weight/day intravenously	5 days	- Significant reduction in complement activation - Significant reduction in myocardial infarct size
Gross *et al.* 2005 [54]	Canine model of myocardial ischemia/reperfusion	DDA 50 mg/kg body weight/day intravenously	2 h or daily for four days	- Significant reduction in myocardial infarct size - Two of three dogs treated for four days had 100% cardiac protection
Gross *et al.* 2006 [36]	Sprague-Dawley rats Left anterior descending coronary artery occlusion/reperfusion	DDA 125 or 500 mg/kg body weight/day orally	7 days	- Astaxanthin loading of myocardium indicating good bioavailability - Trends in lowering of lipid peroxidation products - Significant reduction in myocardial infarct size
Hussein *et al.* 2006 [72]	Spontaneously hypertensive rats	Astaxanthin 5% in olive oil (5 mg/kg/day orally)	7 days	- Significant reduction in nitric oxide end products - Significant reduction in elastin bands in aorta - Significant reduction in wall/lumen arterial ratio in coronary arteries
Aoi *et al.* 2008 [73]	ICR mice	Astaxanthin 0.02% w/w	4 weeks	Astaxanthin increased fat utilization during exercise and prolonged exercise Astaxanthin prevented increase in hexanoyl-lysine modification of CPT I with exercise
Nakao *et al.* 2010 [74]	BALC/c mice	Astaxanthin 0, 0.02, 0.08% orally/day	8 weeks	- No change in blood glutathione concentration - No change in lymphocyte mitochondrial membrane potential - Higher myocardial mitochondrial membrane potential and contractility index
Khan *et al.* 2010 [55]	C57BL/6 mice	CDX-085 500 mg/kg body weight/day	14 days	- CDX-085 administered orally to C57BL/6 mice was associated with presence of free astaxanthin in the plasma, heart, liver and platelets - Mice fed astaxanthin had significantly increased basal arterial blood flow and delay in occlusive thrombosis after endothelial injury - Human umbilical vein endothelial cells and platelets from Wistar-Kyoto rats treated with free astaxanthin has significantly increased release of nitric oxide and decreased peroxynitrite levels
	Human umbilical vein endothelial cells and platelets from Wistar-Kyoto rats			

**Table 2.** Clinical studies investigating the safety, bioavailability and effects of astaxanthin on oxidative stress.

**Study**	**Study population (*n* = subject numbers)**	**Dosage**	**Study design**	**Duration of supplementation**	**Effects of astaxanthin**
Iwamoto *et al.* 2000 [70]	Volunteers (*n* = 24)	Different doses: 1.8, 3.6, 14.4, 21.6 mg/day	Open labelled	2 weeks	- Reduction of LDL oxidation
Osterlie *et al.* 2000 [76]	Middle aged male volunteers (*n* = 3)	100 mg	Open labelled	Single dose	- Astaxanthin taken up by VLDL chylomicrons
Mercke Odeberg *et al.* 2003 [77]	Healthy male volunteers (*n* = 32)	40 mg	Open labelled parallel	Single dose	- Enhanced bioavailability with lipid based formulation
Spiller *et al.* 2003 [78]	Healthy adults (*n* = 35)	6 mg/day (3 × 2 mg tablets/day)	Randomised, double blind, placebo controlled	8 weeks	- Demonstrated safety assessed by measures of blood pressure and biochemistry
Coral-Hinostroza *et al.* 2005 [79]	Healthy adult males (*n* = 3)	10 mg and 100 mg	Open labelled	Single dose or 4 weeks	- C_max_ 0.28 mg/L at 11.5 h at high dose and 0.08 mg/L at low dose - Elimination half life 52 ± 40 h - *z*-isomer selectively absorbed
Karppi *et al.* 2007 [80]	Healthy non-smoking Finnish males (*n* = 40)	8 mg/day	Randomised, double blind, placebo controlled	12 weeks	- Intestinal absorption adequate with capsules - Reduced levels of plasma 12 and 15 hydroxy fatty acids - Decreased oxidation of fatty acids
Parisi *et al.* 2008 [81]	Non-advanced age related macular degeneration (*n* = 27)	4 mg/day	Randomised controlled trial open labelled no placebo	12 months	- Improved central retinal dysfunction in age related macular degeneration when administered with other antioxidants
Miyawaki *et al.* 2008 [82]	Healthy males (*n* = 20)	6 mg/day	Single blind, placebo controlled	10 days	- Decreased whole blood transit time (improved blood rheology)
Rufer *et al.* 2008 [83]	Healthy males (*n* = 28)	5 μg/g salmon flesh (wild *vs.* aquacultured)	Randomised, double blind, placebo controlled	4 weeks	- Bioavailability initially better with ingestion of aquacultured salmon but equivalent at day 28. Isomer (3*S*, 3′*S*) greater in plasma compared with isomer proportion in salmon flesh
Park *et al.* 2010 [84]	Healthy females (*n* = 14)	0, 2, 8 mg/day	Randomised, double blind, placebo controlled	8 weeks	- Decreased plasma 8-hydroxy-2′-deoxyguanosine after week four in those taking astaxanthin. - Lower CRP after week four in those taking 2 mg/day astaxanthin
